# Assessing the Impact of a Men’s Mental Health Peer Support Program Using Participatory Methods

**DOI:** 10.1192/j.eurpsy.2025.828

**Published:** 2025-08-26

**Authors:** R. Whitley

**Affiliations:** 1 Psychiatry, McGill University, Montreal, Canada

## Abstract

**Introduction:**

There is a growing interest in the field of men’s mental health, given that men experience elevated rates of certain mental health outcomes including suicide, substance use disorder and overdoses. Evidence suggests that certain male sub-populations are at particular risk, including fathers who are divorced or separated. This increased risk has been attributed to several factors including painful separation from children, an intense decrease in social support, and a sense of failure and shame associated with marital breakdown. Despite these risks, there is a lack of official services targeted at divorced or separated fathers with mental health issues, with only a few grassroots programs available for this demographic. One such service is a peer support program known as Pères Séparés (seperated fathers). This program is officially accredited by the *ministère de la Santé et des Services sociaux du Québec* to provide psychosocial support including one-on-one peer coaching, weekly support groups and financial/legal advice to divorced or separated men in Montreal.

**Objectives:**

We set out to conduct a project using a method known as participatory video. The aim was to elicit the lived experience of service users, including their experiences within the peer support program, and represent these experiences in a short video that can be used for the education of other peers, health care providers, family members and the general public.

**Methods:**

The project involved creating a workgroup of program service users who were initially trained in basic video-techniques. Workgroup members then interviewed each other about their experiences on camera. All interviews were transcribed, with workgroup members reading the transcripts and distilling prominent themes from these interviews for inclusion in the final video.

**Results:**

The resultant 20 minute video contained three themes (i) the unique struggles of separated fathers including intense loneliness, isolation, depression, and suicidality; (ii) the important of the program in reducing this isolation and providing invaluable social support; and (iii) the rehabilitative role of peer support in providing psychosocial education regarding self-care, social reintegration and pathways to recovery from mental health issues. The video was shown in a series of screenings at targeted settings including health-care, community and educational venues. It was also uploaded to social media where it has been viewed over 5,000 times.

**Conclusions:**

The results imply that such grassroots peer-support programs are very well-situated to help vulnerable fathers in their recovery. Moreover, this program provides a model that can be used as a template elsewhere. Indeed, relevant organizations, funders and service providers could consider creating and implementing similar peer support programs oriented towards seperated fathers. These can benefit such men, their families and society as a whole.

**Disclosure of Interest:**

None Declared

